# Acute human bocavirus 1 infection in child with life-threatening bilateral bronchiolitis and right-sided pneumonia: a case report

**DOI:** 10.1186/s13256-019-2222-5

**Published:** 2019-09-14

**Authors:** Inga Ziemele, Man Xu, Anda Vilmane, Santa Rasa-Dzelzkaleja, Lea Hedman, Klaus Hedman, Maria Söderlund-Venermo, Zaiga Nora-Krukle, Modra Murovska, Dace Gardovska

**Affiliations:** 1 Children’s Clinical University Hospital, Riga, Latvia; 2 Department of Pediatrics Rīga Stradiņš University, Riga, Latvia; 3 Department of Virology, University of Helsinki, Helsinki, Finland; 4 Institute of Microbiology and Virology, Rīga Stradiņš University Riga, Riga, Latvia; 5 Helsinki University Hospital Laboratory Service, Helsinki, Finland

**Keywords:** Human bocavirus 1, Lower respiratory tract infection, Acute bronchiolitis, Children

## Abstract

**Background:**

Human bocavirus 1 is a commonly detected human parvovirus. Many studies have shown human bocavirus 1 as a pathogen in association with acute respiratory tract infections in children. However, because human bocavirus 1 persists in the upper airways for extensive time periods after acute infection, the definition and diagnostics of acute human bocavirus 1 infection is challenging. Until now, detection of human bocavirus 1 exclusively, high viral load in respiratory samples, and viremia have been associated with a clinical picture of acute respiratory illness. There are no studies showing detection of human bocavirus 1 messenger ribonucleic acid in the peripheral blood mononuclear cells as a diagnostic marker for acute lower respiratory tract infection.

**Case presentation:**

We report the case of a 17-month-old Latvian boy who presented in intensive care unit with acute bilateral bronchiolitis, with a history of rhinorrhea and cough for 6 days and fever for the last 2 days prior to admission, followed by severe respiratory distress and tracheal intubation. Human bocavirus 1 was the only respiratory virus detected by a qualitative multiplex polymerase chain reaction panel. For the diagnosis of acute human bocavirus 1 infection, both molecular and serological approaches were used. Human bocavirus 1 deoxyribonucleic acid (DNA) was detected simultaneously in nasopharyngeal aspirate, stool, and blood, as well as in the corresponding cell-free blood plasma by qualitative and quantitative polymerase chain reaction, revealing high DNA-copy numbers in nasopharyngeal aspirate and stool. Despite a low-load viremia, human bocavirus 1 messenger ribonucleic acid was found in the peripheral blood mononuclear cells. For detection of human bocavirus 1-specific antibodies, non-competitive immunoglobulin M and competitive immunoglobulin G enzyme immunoassays were used. The plasma was positive for both human bocavirus 1-specific immunoglobulin M and immunoglobulin G antibodies.

**Conclusions:**

The presence of human bocavirus 1 genomic DNA in blood plasma and human bocavirus 1 messenger ribonucleic acid in peripheral blood mononuclear cells together with human bocavirus 1-specific immunoglobulin M are markers of acute human bocavirus 1 infection that may cause life-threatening acute bronchiolitis.

## Background

Acute lower respiratory tract infections (LRTIs) are responsible for significant morbidity and mortality in infants and young children worldwide. Childhood pneumonia accounts for 16% of all deaths of children under 5 years of age and 7–13% of all community cases are life-threatening and require hospitalization. The most common causes of acute LRTIs with divergent presentation are viruses. Respiratory syncytial virus (RSV) is the most common viral cause of pneumonia in young children [1, 2].

Human bocavirus 1 (HBoV1), the second human pathogenic virus in the family *Parvoviridae*, was discovered in 2005 in nasopharyngeal aspirates (NPAs) of children with acute respiratory tract infection (ARTI) [3]. Since then, HBoV1-associated ARTIs have been reported worldwide [4].

On the one hand, there are studies showing evidence of HBoV1 as a pathogen in association with LRTIs, mainly in children up to 3 years of age [5–10]. On the other hand, HBoV1 has also been found in asymptomatic children [11–14]. These states of affairs at first may seem contradictory; however, HBoV1 has been shown to remain in the nasopharynx for several weeks and even months, thereby causing clinically false polymerase chain reaction (PCR) diagnoses. Moreover, life-threatening and even fatal HBoV1 infections have been reported [15–19]. The definition and diagnosis of acute HBoV1 respiratory tract infection is challenging. Detection of HBoV1 deoxyribonucleic acid (DNA) in blood, messenger ribonucleic acid (mRNA), and viral load assessment in respiratory samples and serology have been recommended as the markers to diagnose active HBoV1 infection [6, 7, 20, 21]. According to the best of our knowledge, there are no studies up to now showing detection of HBoV1 mRNA in the peripheral blood mononuclear cells (PBMCs) as a diagnostic marker for acute HBoV1 infection.

In this study, we report an acute HBoV1 infection in an otherwise healthy child with life-threatening acute bilateral bronchiolitis and right-side pneumonia with detected HBoV1-specific immunoglobulin (Ig) M and DNA in cell-free blood plasma as well as HBoV1 mRNA in PBMC.

## Case report

A 17-month-old Latvian boy was admitted to the Children’s Clinical University Hospital of Riga, Latvia, on the seventh day of illness in January 2015. He presented with a history of rhinorrhea and cough for 6 days and fever (axillar temperature 39.0 °C) for the last 2 days prior to admission. Due to severe respiratory distress, he was immediately transferred from the regional hospital to our intensive care unit.

On admission, his respiratory rate was 44 breaths/minute (reference 20–30), heart rate 146 beats/minute (reference 80–130), oxygen saturation 99% (with an oxygen flow of 5 liters/minute via face mask), and axillary temperature 38.7 °C. Auscultation of his lungs revealed bilateral wheezing and crepitation with severe intercostal and subcostal recessions. The other organ systems were without pathology. Due to the severe respiratory distress, tracheal intubation was performed.

The child had been born full term as the seventh in the family. He had no known underlying illness, history of previous hospitalizations, or severe acute illnesses. He had been fully immunized according to the national immunization scheme.

**Fig. 1:**
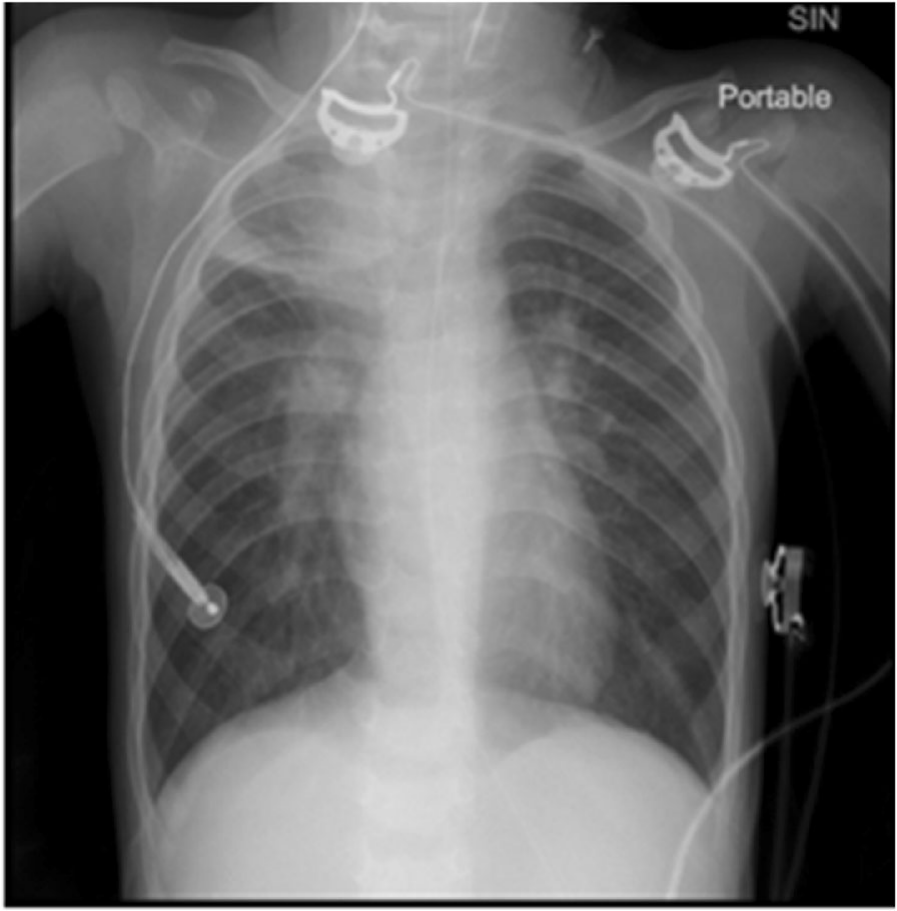
Chest radiograph (anteroposterior view) of the patient on the first day of hospitalization, showing upper right-side pneumonia

On admission, his white blood cell (WBC) count was 30.6 × 10^3^/μL with 66.9% of granulocytes (in absolute numbers 20.6 × 10^3^/μL), hemoglobin 12.4 g/dL, and platelet count 321 × 10^3^/μL. His C-reactive protein (CRP) was 5.09 mg/L. A chest radiograph showed infiltration of the upper lobe of his right lung (Fig. 1).

At the time of admission, a nasopharyngeal swab (NPS) tested negative by direct immunofluorescence (IMAGEN™ OXOID, UK) for antigens of RSV, influenza virus A and B, parainfluenza virus types 1–3, and adenovirus. Bacterial blood cultures were negative. NPA, blood, and stool samples were collected for HBoV1 molecular diagnostics and serology.

NPA tested by qualitative multiplex PCR (Seegene Respiratory Panel, South Korea) was negative for: influenza virus A and B; RSV A and B; flu A types H1, H1pdm09, and H3; adenovirus; enterovirus; parainfluenza virus types 1–4; metapneumovirus; rhinovirus; and coronavirus types NL63, 229E, and OC43. However, the NPA tested by qualitative multiplex PCR was positive for HBoV1. NPA, whole blood with corresponding cell-free blood plasma, and stool samples underwent qualitative PCR for HBoV1 *NS1* DNA, as described [20]. An HBoV1-containing plasmid was used as a positive control in PCR. All these samples were HBoV1 DNA positive. Upon re-examination by quantitative PCR (qPCR) (Human bocavirus genomes, Standard kit, Genesig, Primerdesign Ltd., UK), the copy numbers in NPA and stool were high, 5.7 × 10^5^ per μg DNA in NPA and 1.4 × 10^8^ per μg DNA in stool. The viral load in blood was 21 copies/μg DNA, but in cell-free blood plasma the viral load was under detection level.

**Fig. 2:**
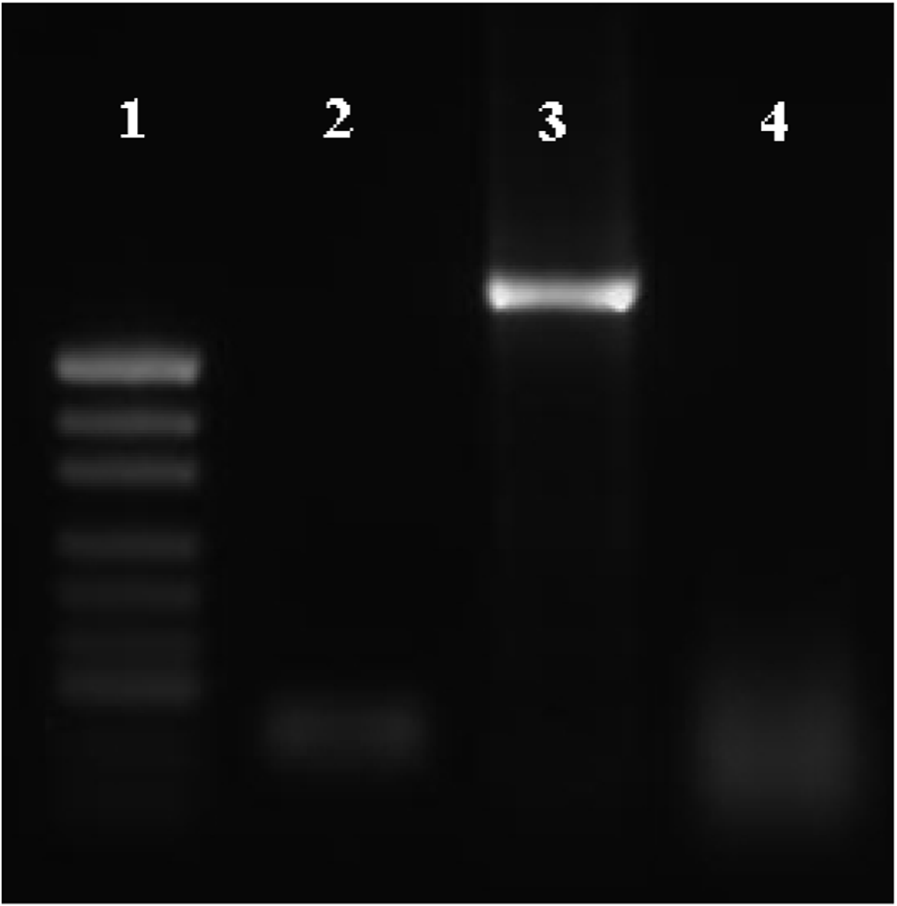
Electrophoretic visualization of amplification products in a 1% agarose gel after polymerase chain reaction targeting *β-actin* gene sequence

To prove that the HBoV1 infection was actively ongoing, HBoV1 transcription in PBMCs was applied. Total ribonucleic acid (RNA) was extracted from PBMCs using TRI Reagent® solution according to the manufacturer’s instructions (Thermo Fisher Scientific, USA). The extracted RNA was quantified spectrophotometrically and analyzed by electrophoresis in a 1% agarose gel. RNA was treated with DNase (TURBO DNA-free™ Kit, Thermo Fisher Scientific, USA) before the synthesis of complementary DNA (cDNA) by the reverse transcriptase (RT) using RevertAid™ First Strand cDNA Synthesis Kit (Thermo Fisher Scientific, USA). The *β-actin* gene sequence was detected by PCR to assess the quality of synthesized cDNA (Fig. 2).

**Fig. 3:**
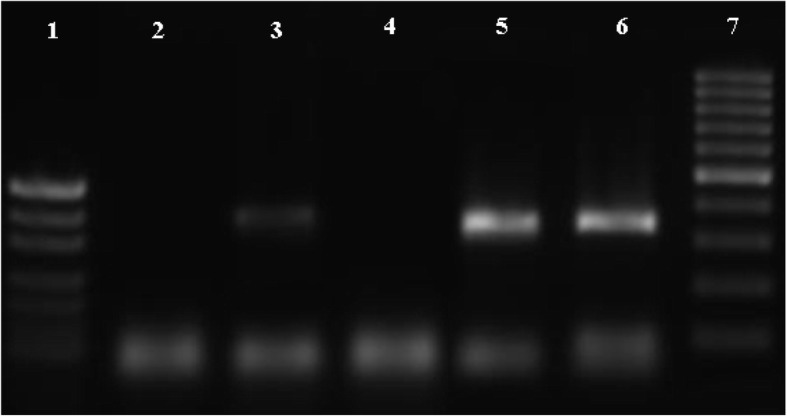
Electrophoretic visualization of amplification products in a 1.7% agarose gel after polymerase chain reaction targeting human bocavirus 1 *NS1* gene. *Legend 1*: *1*. pUC19 DNA/MspI (HpaII) marker; *2*. negative control (molecular biology grade H_2_O); *3*. complementary DNA sample synthesized from DNase treated ribonucleic acid; *4*. DNase treated ribonucleic acid sample without reverse transcriptase step. *Legend 2*: *1*. pUC19 DNA/MspI (HpaII) marker; *2*. DNase treated ribonucleic acid sample without reverse transcriptase step; *3*. complementary DNA sample synthesized from DNase treated ribonucleic acid; *4*. negative control (molecular biology grade H_2_O); *5*., *6*. positive control (human bocavirus 1 plasmid); *7*. GeneRuler 100 bp DNA Ladder

HBoV1-specific cDNA was detected by PCR targeting the HBoV1 *NS1* gene as described by Sloots *et al.*, in 2006, followed by electrophoretic visualization of the amplification products in a 1.7% agarose gel (Fig. 3) [22]. The same DNase-treated RNA sample but without the RT step, served as a negative control in both the β-globin and HBoV1 PCRs to make sure that there was no contamination with DNA.

Biotinylated virus-like particles (VLPs) of the recombinant major capsid protein VP3 were used as antigen in enzyme immunoassays (EIAs) for detection of HBoV1-specific immunoglobulin M (IgM) and immunoglobulin G (IgG) in our patient’s plasma sample [23, 24]. For removal of possible cross-reacting heterologous human bocavirus 2 (HBoV2) and human bocavirus 3 (HBoV3) IgG, non-biotinylated VLPs in competition assays were used as described [24]. Our patient’s plasma sample was positive for both HBoV1-specific IgM and IgG antibodies.

Because of the right lung upper lobe infiltration and increased WBC initially, the child was treated with intravenously administered ceftriaxone 350 mg twice a day for 7 days and per-oral oseltamivir 30 mg twice a day (due to influenza season). Oseltamivir was discontinued after 3 days due to the negative influenza virus A and B antigen findings. Extubated on day 3, our patient was brought to the Department of Paediatrics, where intravenously administered ceftriaxone was continued, inhalations via nebulizer with salbutamol and budesonide were begun and pulmonary rehabilitation started. During the next 10 days, the child’s general condition improved, his body temperature was normal, lung sounds were without the pathology, and no additional oxygen was needed. During the hospitalization, poor weight gain was observed for our patient; therefore, additional diagnostic tests were done and his hospitalization length increased. On day 17 of hospitalization, he developed a new episode of fever for 2 days. The second NPS tested negative for RSV, and influenza virus A and B; however, self-limiting viral upper respiratory tract infection was suspected and he was treated with intravenously administered rehydration and ibuprofen 70 mg for these 2 days. Due to the very low socioeconomic status of the family, he was kept in the hospital mainly for observation, although his general condition was good. On day 30 he developed a new episode of fever, cough, and wheezing lasting 6 days. In this episode, LRTI was diagnosed based on the clinical symptoms and he was treated with nebulized salbutamol and budesonide.

After 46 days of hospitalization he recovered completely from HBoV1-associated acute bilateral bronchiolitis with right-side pneumonia and a subsequent hospital-acquired upper and LRTI and was discharged.

## Discussion

HBoV1 is frequently found in respiratory tract samples collected from hospitalized children with a peak age up to 24 months [24–26]. By PCR it appears as the third most common pathogen next to RSV and rhinovirus in young children presenting with acute bronchiolitis and wheezing [4, 27, 28]. Furthermore, serological studies have revealed that HBoV1 acute infections occur most often in early childhood, with HBoV1 IgG seroconverting at a median age of 1.9 years and reaching a seroprevalence of 80% by 6 years of age [24]. Several clinical cases have shown severe or even life-threatening respiratory tract diseases due to HBoV1 infection in children [10, 15–19, 29]. However, it should be kept in mind that, due to HBoV1 long-term persistence in the upper airways, the virus is frequently co-detected with other respiratory pathogens. The diagnosis should therefore not be based only on qualitative PCR for HBoV1 DNA in respiratory samples [6, 20, 30]. Detection of HBoV1 DNA in blood, mRNA, and viral load assessment in airway samples and serology have been recommended as better tools for diagnostics to separate active HBoV1 infection from asymptomatic virus shedding [6, 7, 20, 21].

In this report, we present a case of a 17-month-old boy with typical symptoms of acute bilateral bronchiolitis: initially rhinorrhea and cough followed by difficulty in breathing and typical findings in auscultation, including wheezing and crepitation. Fever occurred for 2 days before hospitalization and his WBC was high with absolute granulocytosis. Because the latter findings pointed to complications, chest radiography was performed and right-sided pneumonia confirmed. The onset of the clinical course and normal range CRP value, were compatible with a viral LRTI.

NPA tested by multiplex PCR was positive only for HBoV1 DNA, while 18 other respiratory viruses, including those which often cause severe bronchiolitis in this age group (RSV, rhinovirus, and human metapneumovirus), were negative. It was not surprising that HBoV1 DNA was simultaneously found by PCR in all samples including stool. Viral passage through the gastrointestinal tract due to swallowing of respiratory secretions in patients with ARTIs has been suggested [9]. By qPCR, a high viral load was detected in NPA and stool. Detection of HBoV1 exclusively, high HBoV1 DNA load in respiratory samples, and viremia are associated with a clinical picture of acute LRTI [6, 7]. However, in some other cases the viral DNA has remained detectable in blood for a prolonged time [15, 16]. In our patient, HBoV1 PCR was also positive in cell-free blood plasma, indicating that the virus was freed from the cells and that he had HBoV1 viremia. Diagnosis of acute HBoV1 infection is shown also by the presence of circulating HBoV1-specific IgM. Moreover, to verify that the current illness was in fact caused by acute HBoV1 infection, we looked for and found HBoV1 mRNA in PBMCs, verifying RNA transcription. To the best of our knowledge this is the first time HBoV1 mRNA has been detected in PBMCs, suggesting active replication in PBMCs. Existing data support that the detection of HBoV1 mRNA, at least in NPA, is more accurate than that of HBoV1 DNA, in the diagnosis of active infection [21, 31].

## Conclusions

HBoV1 infection may cause life-threatening acute bronchiolitis, as this pediatric case demonstrates. The diagnosis of acute HBoV1 infection was proved by the presence of HBoV1-specific IgM and DNA in cell-free blood plasma as well as HBoV1 mRNA in PBMCs, whereas no other viruses or bacteria were found by PCR and culture, respectively.

## Data Availability

Data available by request to: inga.ziemele@gmail.com.

## Abbreviations

ARTI: Acute respiratory tract infection
cDNA: Complementary DNA
CRP: C-reactive protein
EIA: Enzyme immunoassay
HBoV1: Human bocavirus 1
HBoV2: Human bocavirus 2
HBoV3: Human bocavirus 3
Ig: Immunoglobulin
LRTI: Lower respiratory tract infection
mRNA: Messenger ribonucleic acid
NPA: Nasopharyngeal aspirate
NPS: Nasopharyngeal swab
PBMCs: Peripheral blood mononuclear cells
PCR: Polymerase chain reaction
qPCR: Quantitative polymerase chain reaction
RNA: Ribonucleic acid
RSV: Respiratory syncytial virus
RT: Reverse transcriptase
VLPs: Virus-like particles
WBC: White blood cell
